# Spatiotemporal analysis of putative notochordal cell markers reveals CD24 and keratins 8, 18, and 19 as notochord‐specific markers during early human intervertebral disc development

**DOI:** 10.1002/jor.23205

**Published:** 2016-03-07

**Authors:** Ricardo Rodrigues‐Pinto, Andrew Berry, Karen Piper‐Hanley, Neil Hanley, Stephen M. Richardson, Judith A. Hoyland

**Affiliations:** ^1^ Centre For Tissue Injury and Repair, Institute of Inflammation and Repair, Faculty of Medical and Human SciencesUniversity of ManchesterStopford Building, Oxford RoadManchesterM13 9PTUnited Kingdom; ^2^ Department of OrthopaedicsCentro Hospitalar do Porto—Hospital de Santo AntónioLargo Prof. Abel SalazarPorto4099‐001Portugal; ^3^ Centre for Endocrinology and Diabetes, Institute of Human Development, Faculty of Medical and Human SciencesUniversity of ManchesterAV Hill Building—3rd Floor, Oxford RoadManchesterM13 9PTUnited Kingdom; ^4^ NIHR Manchester Musculoskeletal Biomedical Research UnitManchester Academic Health Science CentreManchesterUnited Kingdom

**Keywords:** intervertebral disc degeneration, notochordal cells, nucleus pulposus, ontogeny, phenotype

## Abstract

In humans, the nucleus pulposus (NP) is composed of large vacuolated notochordal cells in the fetus but, soon after birth, becomes populated by smaller, chondrocyte‐like cells. Although animal studies indicate that notochord‐derived cells persist in the adult NP, the ontogeny of the adult human NP cell population is still unclear. As such, identification of unique notochordal markers is required. This study was conducted to determine the spatiotemporal expression of putative human notochordal markers to aid in the elucidation of the ontogeny of adult human NP cells. Human embryos and fetuses (3.5–18 weeks post‐conception (WPC)) were microdissected to isolate the spine anlagens (notochord and somites/sclerotome). Morphology of the developing IVD was assessed using hematoxylin and eosin. Expression of keratin (KRT) 8, KRT18, KRT19, CD24, GAL3, CD55, BASP1, CTGF, T, CD90, Tie2, and E‐cadherin was assessed using immunohistochemistry. KRT8, KRT18, KRT19 were uniquely expressed by notochordal cells at all spine levels at all stages studied; CD24 was expressed at all stages except 3.5 WPC. While GAL3, CD55, BASP1, CTGF, and T were expressed by notochordal cells at specific stages, they were also co‐expressed by sclerotomal cells. CD90, Tie2, and E‐cadherin expression was not detectable in developing human spine cells at any stage. This study has identified, for the first time, the consistent expression of KRT8, KRT18, KRT19, and CD24 as human notochord‐specific markers during early IVD development. Thus, we propose that these markers can be used to help ascertain the ontogeny of adult human NP cells. © 2016 The Authors. *Journal of Orthopaedic Research* Published by Wiley Periodicals, Inc. J Orthop Res 34:1327–1340, 2016.

The search for novel therapies for intervertebral disc (IVD) degeneration has motivated an increased interest in the understanding of the native nucleus pulposus (NP) cell phenotype and the ontogeny of its component cells to guarantee that implanted cells have the correct phenotype to ensure adequate function. While the human developing NP is composed of large vacuolated notochordal cells, the adult NP contains small non‐vacuolated cells whose ontogeny, despite lineage tracing studies in mice,^1^, ^2^ is still a subject of debate. It is unclear whether the original population of notochordal cells differentiates into the smaller NP cells present within adult tissue, dies to be replaced by cells migrating from adjacent tissues or both. To clarify this controversy and, since cell size and morphology differences are not uncommon in cells with common ancestry,^3^ specific molecular markers for human notochordal cells are needed.

Several studies have investigated the NP cell phenotype in rats,^4^, ^5^, ^6^ dogs,^7^ cows,^8^ and since the NP phenotype differs between species,^9^ in humans.^10^, ^11^, ^12^ Interestingly, some of the genes identified in the human adult NP had previously been identified within larger, notochordal cells of bovine IVD.^8^ These studies, however, could not clarify how specific to notochordal cells those genes were and, therefore, how indicative of notochordal ontogeny they could be.

To adequately clarify the ontogeny of the cells populating the adult NP, it is fundamental to understand IVD development and to identify unique notochordal cell markers that may allow the identification of notochord‐derived cells in humans, even after a morphological change or differentiation. Studies have investigated the role of notochordal cells in IVD development in rats^1^, ^6^, ^13^, ^14^; however, only a limited number of studies have investigated the notochordal cell phenotype in humans.^15^, ^16^, ^17^, ^18^ Unfortunately, these studies have either had access to very limited number of samples and/or have focused on the investigation of the expression of extracellular matrix proteins. These studies, although informative regarding the microenvironment and the physicochemical characteristics of the developing IVD, do not elucidate the phenotype of the developing notochordal cells, or provide unique notochordal markers and, hence, do not clarify the ontogeny of adult human NP cells.

A recent review has provided a comprehensive list of markers previously associated with the phenotype of notochordal cells in animals or with the phenotype of immature human NP cells^19^ and highlighted keratin (KRT) 8,^20^, ^21^ KRT18,^10^, ^12^, ^20^, ^21^ KRT19,^10^, ^12^, ^20^, ^21^ brachyury (T),^6^ galectin 3 (GAL3),^22^ CD24,^23^ CD55,^21^ brain abundant membrane attached signal protein (BASP1),^21^ connective tissue growth factor (CTGF)^24^ and E‐Cadherin (E‐Cad)^20^ as putative notochordal/ immature NP markers, Tie2^25^ as a NP progenitor cell marker and CD90^6^, ^23^ as negative NP marker. However, to date, the spatiotemporal expression of these markers in the human developing spine and notochord has not been analyzed and, therefore, their suitability as unique human notochordal cell markers has not been assessed. The identification of such markers would help researchers to trace the fate of notochordal cells during human IVD development, maturation, and degeneration and to understand if, despite having acquired a different morphology, notochord‐derived cells persist in the adult human NP. To date, however, such studies have not been conducted and this is a major limitation in the field.

This study was, therefore, conducted with the objective of identifying human embryonic and fetal notochordal cell‐specific markers that could aid in the understanding of the notochordal NP cell development and phenotype and hence help elucidate the ontogeny of the cells populating the adult NP.

## METHODS

### Sample Acquisition and Staging

Human embryonic and fetal samples (Table 1) were obtained with ethical approval from the local research ethics committee, Ref. No: 08/H1010/28 Early Pregnancy Tissue Collection) and with full informed consent following medical or surgical pregnancy termination). Embryonic staging was performed according to the Carnegie classification^26^ and converted to weeks post‐conception (WPC) and fetal staging was estimated by hand and foot length measurements.

**Table 1:** Developmental Stages of the Human Samples Used for Hematoxylin and Eosin (H&E) and Immunohistochemistry (IHC) Staining

Sample ID	WPC (Carnegie Stage)	Used for
1	3.5 (CS10)	H&E
2	5.5 (CS16)	H&E and IHC
3	6	H&E
4	7	H&E and IHC
5	7	H&E
6	7.5	H&E and IHC
7	7.5	H&E and IHC
8	7.5	H&E
9	7.5	H&E
10	8	H&E
11	8	H&E
12	8	H&E
13	8.5	H&E
14	8.5	H&E and IHC
15	9	H&E and IHC
16	9.5	H&E
17	9.5	H&E and IHC
18	10	H&E
19	10	H&E and IHC
20	10	H&E
21	10.5	H&E and IHC
22	11	H&E and IHC
23	11.5	H&E and IHC
24	12	H&E and IHC
25	12	H&E and IHC
26	12.5	H&E and IHC
27	13	H&E and IHC
28	14	H&E and IHC
29	14	H&E
30	17	H&E and IHC
31	18	H&E and IHC

### Human Embryonic and Fetal Spine Dissection, Processing, and Preparation for Immunohistology

Samples were processed within 2–4 h of acquisition. Younger embryos (3.5–5.5 WPC) were whole‐mounted. In embryos older than 5.5 WPC and in fetuses (8–18 WPC), the whole spine (vertebrae and intervertebral discs) was dissected from the adjacent tissues. Dissection was performed under sterile conditions, using microsurgical instruments and a stereomicroscope (Stemi 2000, Carl Zeiss®, Dublin, CA). Briefly, the spines and their adjacent tissues (ligaments, ribs, and spinal cord) were carefully dissected from the embryo/fetus and transferred to a Petri dish containing phosphate buffered saline (PBS); then, using microsurgical forceps and scissors, the ribs (at their costovertebral joints) and the spinal cord, were gently separated from the spine; third, the anterior and posterior longitudinal ligaments were gently separated from the spine; finally, the resulting whole fetal spine containing the vertebrae and IVDs was washed in PBS.

Whole fetal spines were fixed immediately after harvest in 4% paraformaldehyde (Sigma‐Aldrich®, Irvine, UK, 36148) in PBS for 24 h, after which they were decalcified for 3 days in EDTA (20% EDTA pH 7.4 [Tennaquest®, Manchester, UK]) and then washed in running water for 1 day. Decalcified fetal spines were processed overnight, embedded in paraffin wax and cut into 5 µm sections with a microtome. The 3.5 WPC fetal specimen was sectioned transversally while all other specimens were sectioned longitudinally along the whole spinal length.

Sections were mounted on positively charged slides (Thermo Scientific®, Hemel, Hempstead, UK, J1800AMNZ), de‐paraffinized in xylene (Fisher Scientific®, Indianapolis, IN, X/0200/17) (3 changes × 5 min) and re‐hydrated through four changes industrial methylated spirits (IMS, Fisher Scientific®, M/4450/17) (2 min each) to water.

### Histology and Immunohistochemistry

For morphological analysis of the developing spine, slides were stained in Mayer's hematoxylin & eosin (H&E) according to standard published protocols.

Protein expression of KRT8, KRT18, KRT19, CD24, GAL3, CD55, BASP1, CTGF, T, CD90, Tie2, and E‐cad was assessed using immunohistochemistry utilizing the Avidin–Biotin Complex method. For the antibodies where enzyme‐only antigen retrieval methods were used, endogenous peroxidase blockade was performed prior to the antigen retrieval and for those where heat antigen‐retrieval methods were used, antigen retrieval was performed prior to blocking endogenous peroxadises (see Table 2 for antigen retrieval methods). Endogenous peroxidase blockade was performed by immersing slides in 100% IMS containing 0.3% (v/v) hydrogen peroxide and 25 mM HCl for 30 min at room temperature and antigen retrieval as specified in Table 2. After antigen retrieval and endogenous peroxidase blockade, non‐specific binding sites were blocked with 25% (v/v) goat serum (Sigma–Aldrich, G9023) in 1% w/v BSA (Sigma‐Aldrich, A9647) in TBS (except for Gal‐3 and KRT8 where 2% and 5% w/v BSA in TBS were used, respectively). Following blocking, slides were stained overnight at 4°C (for KRT8, KRT18, and KRT19, Galectin‐3, CD55, and BASP1) or for 2 h at room temperature (for CD24, CTGF, Tie2, CD90, E‐Cad, and T) with primary antibodies diluted in 1% v/w BSA in TBS (2% and 5% for Gal‐3 and KRT8, respectively). Primary antibodies were disclosed by incubating for 30 min with biotin conjugated secondary antibodies (1.33 μg/ml goat anti‐mouse IgG (Santa Cruz Biotechnology®, Heidelberg, Germany, sc‐3795) for primary antibodies raised in mice and 1.33 μg/ml goat anti‐rabbit IgG (Santa Cruz Biotechnology®, sc‐3840) for primary antibodies raised in rabbit). Amplification was performed by incubating 30 min with ABC‐Amplification reagent (Vectastain®, Burlingame, CA) and detection of the Avidin–Biotin complex was performed by incubating for 18 min with 3, 3′‐diaminobenzidine (DAB) (Sigma–Aldrich®, D5905‐50TAB). Nuclei were counterstained in Mayer's hematoxylin for 90 s. Between each of these steps (antigen retrieval, blocking, primary antibody incubation, secondary antibody incubation, amplification, and detection) slides were immersed three times in TBS (0.5 M Tris Base (Fisher Bioreagent®, Pittsburg, KS, BP152‐1), 9% w/v NaCl (Fisher Chemical®, Indianapolis, IN, S/3160/65), pH 7.6) for 5 min. After staining, sections were dehydrated in four changes of IMS (2 min each), cleared in three changes of xylene (5 min each) and mounted with a coverslip in mounting medium (Shand Consul‐Mount, Thermo Scientific®, 9990440).

**Table 2:** Immunohistochemistry Methodology: Details of the Antibodies and Antigen Retrieval Methods Used for Each Marker

Marker	Antibodies (Optimized Concentration, Clonality, Manufacturer, Catalog Number)	Antigen Retrieval Method
KRT8	0.5 μg/ml mouse monoclonal anti‐KRT8 IgG1 (Zytromed Systems, 603–2156)	Pepsin + Pronase^a^
KRT18	0.338 μg/ml mouse monoclonal Anti‐KRT18 IgG1 (DakoCytomation M7010)	Heat TrisEDTA^b^
KRT19	0.016 μg/ml mouse monoclonal anti‐KRT19 IgG1 (DakoCytomation, M0888)	Heat TrisEDTA^b^
CD24	0.20 mg/ml mouse monoclonal anti‐CD24 IgG1 (Abcam, ab31622)	Heat Citrate^c^
GAL3	4 μg/ml rabbit polyclonal anti‐Galectin‐3 IgG (Santa Cruz Biotechnology, sc‐20157)	Pepsin + Pronase^a^
CD55	25 μg/ml mouse monoclonal anti‐CD55 IgM (Sigma‐Aldrich, SAB4700249)	Heat TrisEDTA^b^
CTGF	1 μg/ml mouse monoclonal anti‐CTGF IgG1 (R&D Systems, MAB660)	Heat Citrate^c^
BASP1	0.67 μg/ml rabbit polyclonal anti‐BASP1 IgG (Santa Cruz Biotechnology, sc‐66994)	Heat TrisEDTA^b^
Tie2	4 μg/ml mouse monoclonal anti‐Tie2 IgG1 (Novus Biologicals, NB110‐60986)	Proteinase K^d^
CD90	0.184 μg/ml rabbit monoclonal anti‐CD90 IgG (Abcam, ab133350)	Heat Citrate^c^
E‐Cad	1.3 μg/ml mouse monoclonal anti‐E‐Cad IgG1 (Abcam, ab1416)	Heat Citrate^c^
T	2 μg/ml rabbit polyclonal anti‐Brachyury IgG (Abcam, ab 20680)	Heat Citrate^c^

^a^ Ten minutes incubation in 0.25% Pepsin in HCl + 10 min incubation in 0.1% pronase in TBS pH = 7.2.

^b^ Heat incubation (10 min steamer + 10 min bench) in TrisEDTA buffer (10 mM Tris, 1 mM EDTA, pH 8.0).

^c^ Heat incubation (10 min steamer + 10 min bench) in citrate buffer (10 mM citric acid, pH 6.0).

^d^ Fifteen minutes incubation in 20 μg/ml proteinase K in TBS.

Optimal primary antibody concentrations were optimized using human fetal sections and, for antibodies with negative staining in the fetal spine, using an appropriate positive control tissue. To exclude non‐specific staining, additional fetal samples were also stained with isotype immunoglobin (IgG) controls at the same protein concentration as the primary antibodies. Unless otherwise specified, all procedures were performed at room temperature and incubations were performed in a wet box to prevent the slides from drying out.

Staining was visualized using a light microscope (Dialux 20EB, Leitz®) and captured using the Pannoramic 250 Flash II digital slide scanner (3DHistech®, Budapest, Hungary) and visualized using the Pannoramic Viewer software (3DHistech®).^27^ For each antibody, sufficient images were chosen to depict the staining along the developmental stages analyzed.

## RESULTS

### Morphology

**Figure 1:**
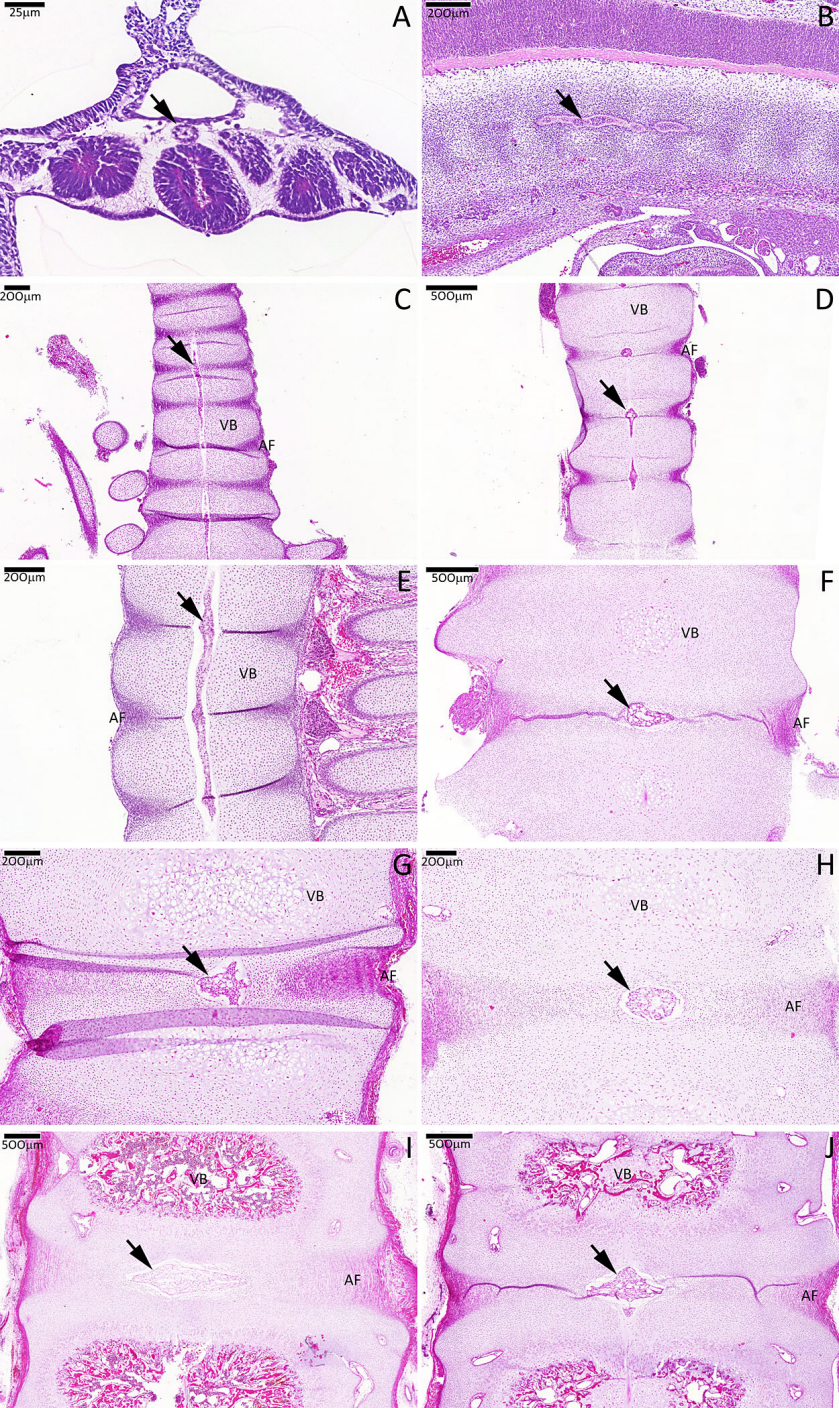
H&E staining of a cohort of developing spines. The notochord develops as a rod‐like centrally located structure formed by large and vacuolated notochordal cells (arrows) and surrounded by somites which will later become sclerotomal cells. During the analyzed stages, the notochord involutes to become localized to the central IVD region (NP anlagen) and sclerotomal cells adopt a segmented pattern; densely organized sclerotomal regions will form the developing AF. Sclerotomal cells in the adjacent regions have a round morphology and will later form the VB. (A) CS10, (B) CS16, (C) 7 WPC, (D) 8 WPC, (E) 9 WPC, (F) 10 WPC, (G) 12 WPC, (H) 14 WPC, (I) 17 WPC, (J) 18 WPC.

Large vacuolated notochordal cells were identified in all specimens. In the earlier stage (3.5 WPC) notochordal cells were organized side‐by‐side forming a cylindrical midline epithelioid‐like structure, the notochord. The notochord was positioned anteriorly to the neural tube and had a row of somites on each side. With embryonic growth (5.5–8 WPC), the notochord and somites elongated along the embryo axis; somite cells migrated laterally (becoming dermatomyotomal cells, dermis, and muscle precursors) and centrally, toward the midline (becoming sclerotomal cells) while adopting a segmented morphology pattern. Segments with higher cell density (precursors of the AF in the IVD region) alternated with less densely organized segments (precursors of the vertebral body). After entering the foetal stage (eighth WPC) the notochord within the less densely organized sclerotomal segments started to involute and occupied a wider midline area within the adjacent segments (IVD anlagens); here, its vacuoles had become larger. This involution was completed after the 10th WPC, with notochordal cells completely absent from VB anlagens and just remnants of the notochordal sheath being present in this region; at this stage and in all stages thereafter, notochordal cells were restricted to the IVD segments. Sclerotomal cells within the IVD anlagen region had a lamellar organization, characteristic of AF cells, and encircling the central notochordal NP anlagen; those in the adjacent VB segments had a chondrocyte‐like morphology and with hypertrophic chondrocytes in its center (Fig. 1).

### Immunohistochemical Identification of Developmental Markers

No differences in staining intensity were noted for any of the antibodies analyzed in the different spinal levels (cervical, thoracic, and lumbar) at each specific developmental stage. All isotype control sections were routinely negative.

#### Notochord‐Specific Markers

**Figure 2:**
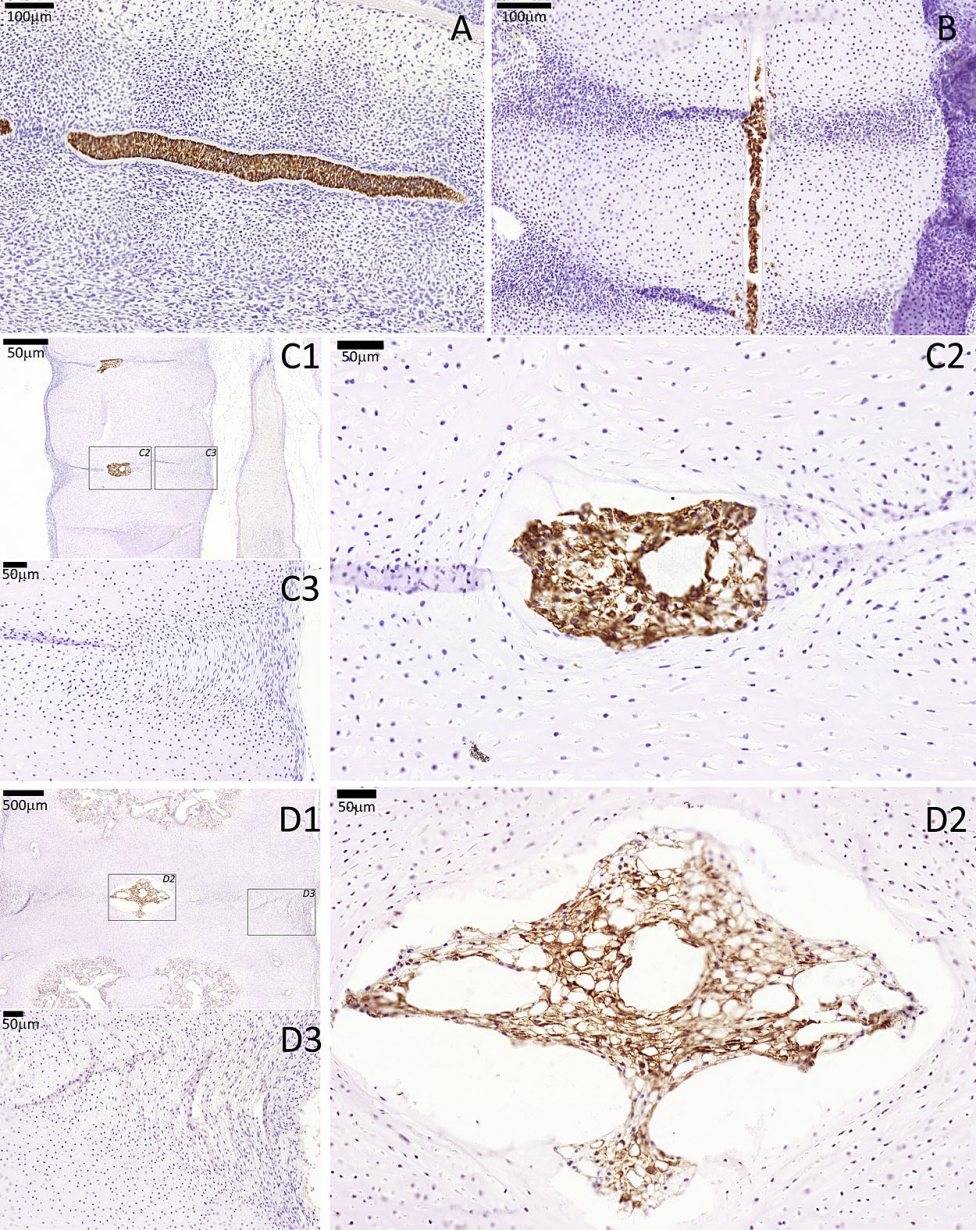
KRT18 immunostaining of a cohort of developing spines showing notochord‐specific expression of this marker. No KRT18 expression was seen in the surrounding sclerotomal AF and VB cells. (A) CS16, (B) 7 WPC, (C) 10 WPC, (D) 18 WPC. For stages 10 and 18 WPC, two higher magnifications are highlighted (squares), one that is centered to the developing notochordal NP (C2 and D2) and the other that is centered to the developing sclerotomal AF (C3 and D3).

KRT8, KRT18, and KRT19 were specifically expressed by all notochordal cells in all developmental stages analyzed (3.5–18 WPC); staining for the three keratins was localized to the cytoplasm and around, but not inside, the vacuoles. Somite (3.5 WPC) and sclerotomal (5.5–18 WPC) cells in the developing AF and VB did not express KRT8, KRT18, or KRT19 in any of the analyzed stages (Fig. 2 and Supplementary Figs. S1 and S2).

**Figure 3:**
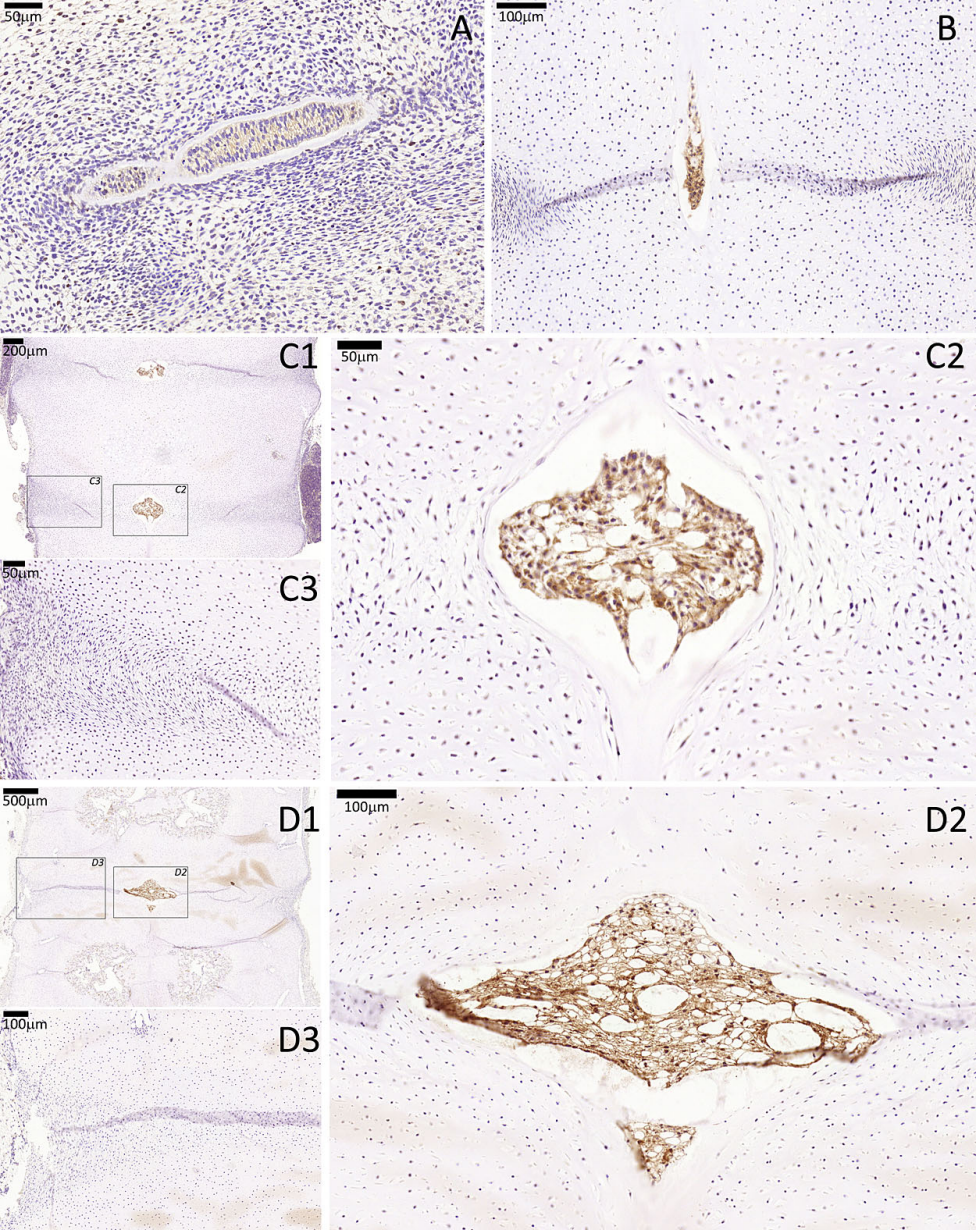
CD24 immunostaining of a cohort of fetal spines showing notochord‐specific expression of this marker between CS16 and 18 WPC. No CD24 expression was seen in the surrounding sclerotomal cells in the developing AF and VB in all analyzed stage. (A) CS16, (B) 8 WPC, (C) 10 WPC, (D) 18 WPC. For stages 10 and 18 WPC, two higher magnifications are highlighted (squares), one that is centered to the developing notochordal NP (C2 and D2) and the other that is centered to the developing sclerotomal AF (C3 and D3).

CD24 was not expressed by notochordal or somite cells in the 3.5 WPC sample. Between 5.5–18 WPC, however, CD24 was specifically expressed in the extracellular membrane of all notochordal cells. No sclerotomal staining was seen in any of the developmental stages analyzed (Fig. 3).

#### Protein Markers Demonstrating Variable Expression With Developmental Stages

**Figure 4:**
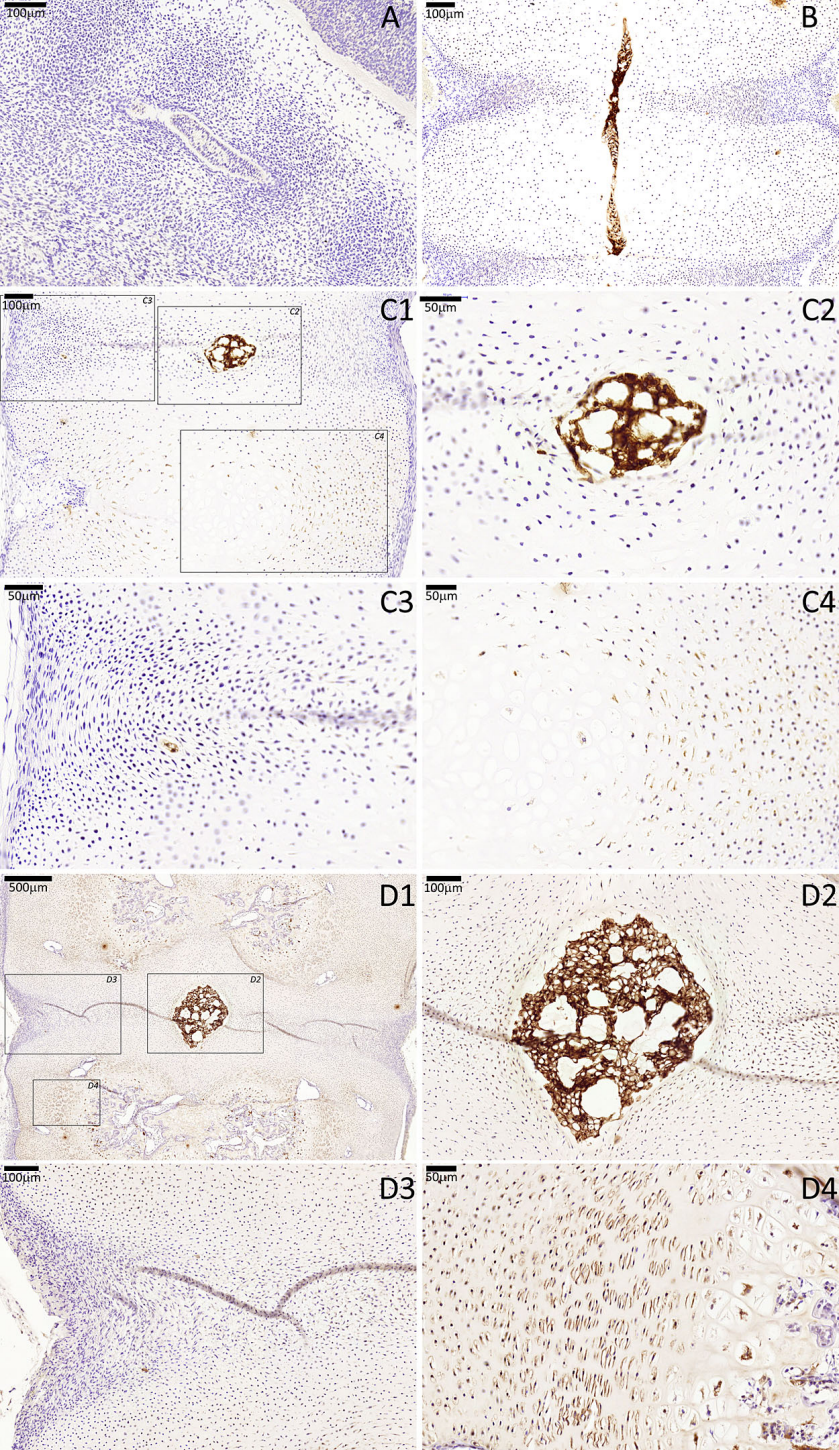
GAL3 immunostaining of a cohort of fetal spines. GAL‐3 was notochord‐specific between 7 and 9 WCP after which it became co‐expressed by sclerotomal VB cells. (A) CS16, (B) 7.5 WPC, (C) 11 WPC, (D) 18 WPC. For stages 11 and 18 WPC, three higher magnifications are highlighted (squares), one that is centered to the developing notochordal NP (C2 and D2), other that is centered to the developing sclerotomal AF (C3 and D3) and another that is centered to the developing VB (C4 and D4).

GAL3 was not expressed by notochordal or somite cells in the 3.5 and 5.5 WPC samples. Between 7 and 18 WPC, however, GAL3 was expressed in the cytoplasm of all notochordal cells. The expression was notochord‐specific between 7 and 9 WPC but was co‐expressed in the cytoplasm of sclerotomal VB cells between 10 and 18 WPC; no expression was noted in the sclerotomal AF cells at any stages analyzed (Fig. 4).

**Figure 5:**
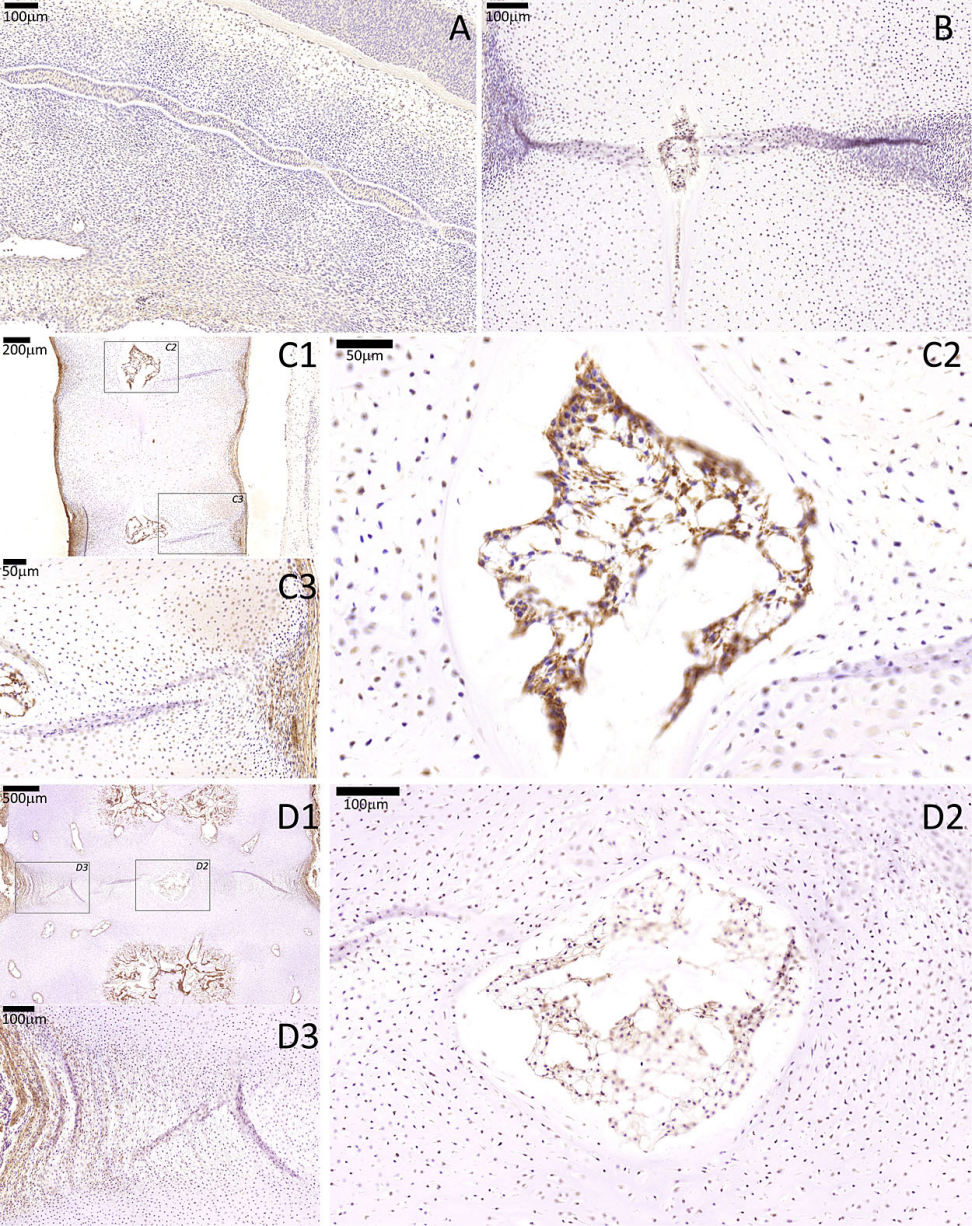
CD55 immunostaining of a cohort of developing spines. CD55 was notochord‐specific between CS16 and 10 WPC, after which it became co‐expressed by sclerotomal cells in the developing AF. (A) CS16, (B) 7.5 WPC, (C) 12 WPC, (D) 18 WPC. For stages 12 and 18 WPC, two higher magnifications are highlighted (squares), one that is centered to the developing notochordal NP (C2 and D2) and the other that is centered to the developing sclerotomal AF (C3 and D3).

CD55 was not expressed by notochordal or somite cells in the 3.5 and 5.5 WPC specimens. Between 7 and 9 WPC, CD55 was specifically expressed on the extracellular membrane of all notochordal cells and in these stages sclerotomal cells did not express CD55. However, after 10 WPC, CD55 became co‐expressed by the sclerotomal cells in the developing AF; sclerotomal cells in the VB anlagen never expressed CD55 (Fig. 5).

**Figure 6:**
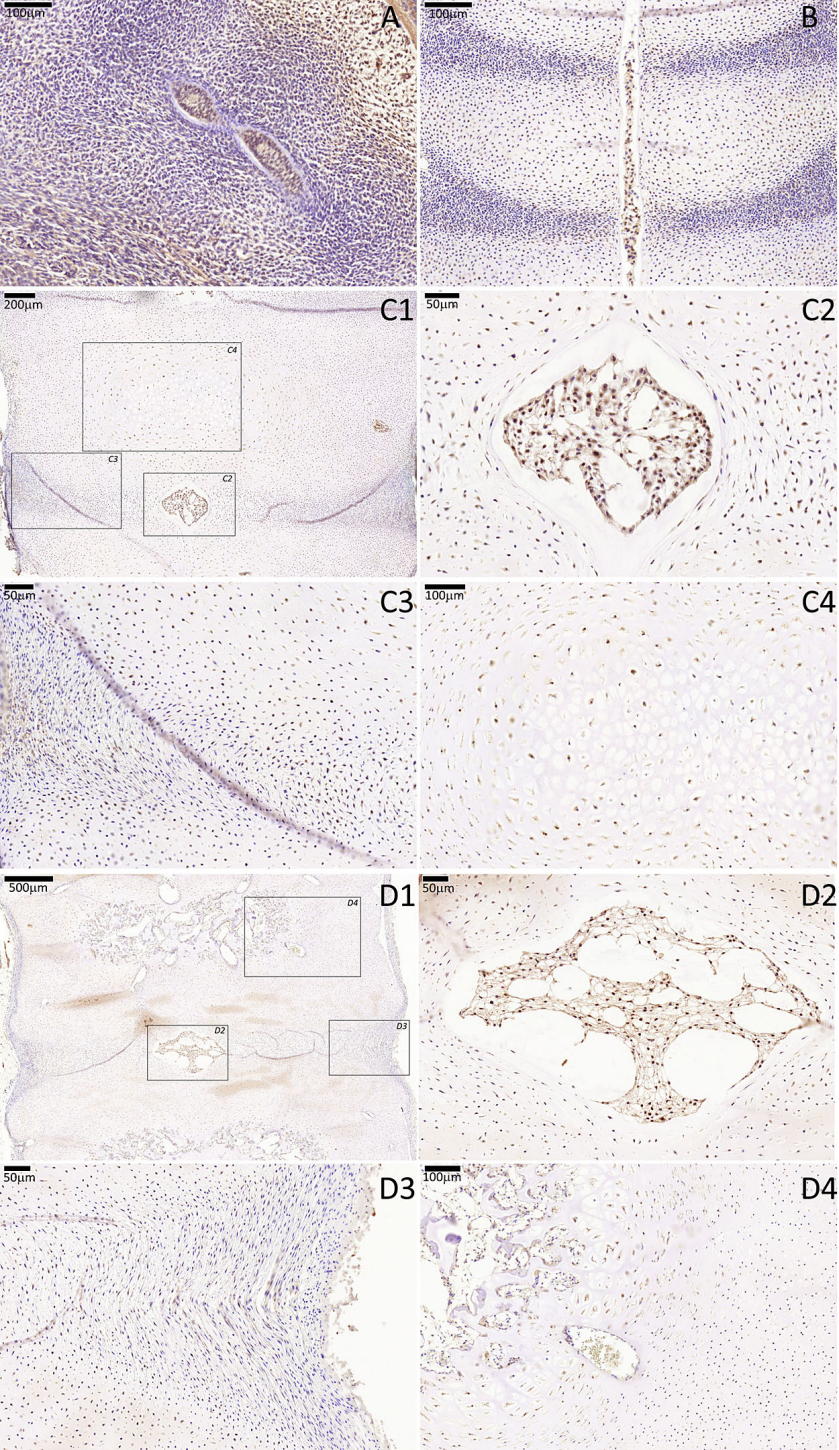
T immunostaining of a cohort of developing spines showing notochord and sclerotomal (VB anlagen) co‐expression between CS16 and 18 WPC. (A) CS16, (B) 7 WPC, (C) 10 WPC, (D) 18 WPC. For stages 10 and 18 WPC, three higher magnifications are highlighted (squares), one that is centered to the developing notochordal NP (C2 and D2), other that is centered to the developing sclerotomal AF (C3 and D3) and another that is centered to the developing VB (C4 and D4).

T was notochord‐specific in the 3.5 WPC specimen. In the 5.5 WPC specimen, T was expressed by notochordal and sclerotomal cells. Between 6 and 18 WPC, T was expressed by all notochordal and sclerotomal cells in the VB anlagens; no expression was seen in the AF anlagens at any stage analyzed (Fig. 6).

**Figure 7:**
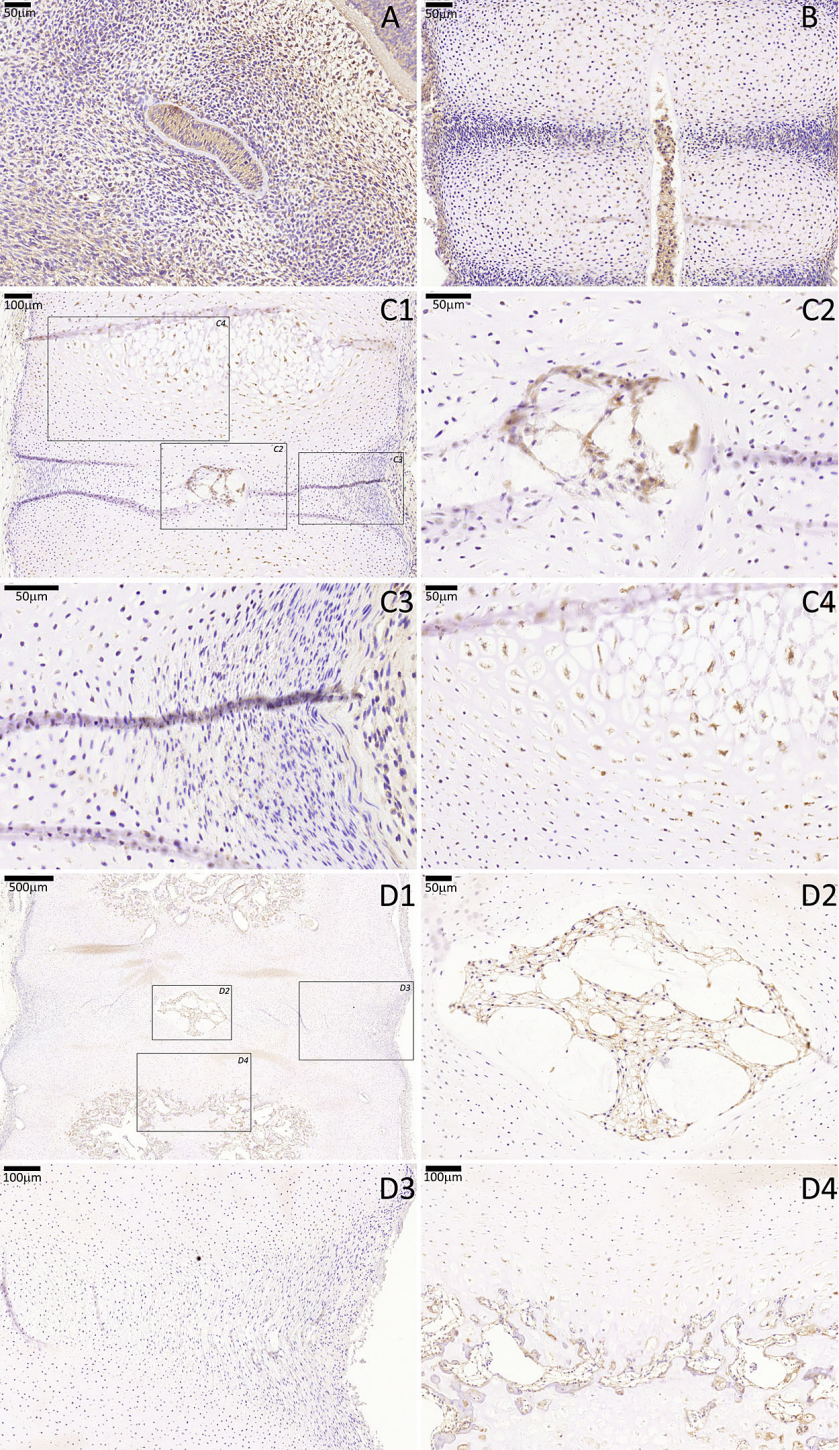
CTGF immunostaining of a cohort of fetal spines showing notochord and VB co‐expression between CS16 and 18 WPC. (A) CS16, (B) 7 WPC; (C) 11 WPC, (D) 18 WPC. For stages 11 and 18 WPC, three higher magnifications are highlighted (squares), one that is centered to the developing notochordal NP (C2 and D2), other that is centered to the developing sclerotomal AF (C3 and D3) and another that is centered to the developing VB (C4 and D4).

In the 3.5 and 5.5 WPC specimens, CTGF was not expressed by notochord or somite/sclerotomal cells. Between 6 and 18 WPC, CTGF expression was found in all notochordal and sclerotomal cells in the developing VB. The intensity of VB staining was weak in all stages and that of notochordal cells was weak between 11 and 17 WPC. Sclerotomal AF cells did not express CTGF at any stage analyzed (Fig. 7).

**Figure 8:**
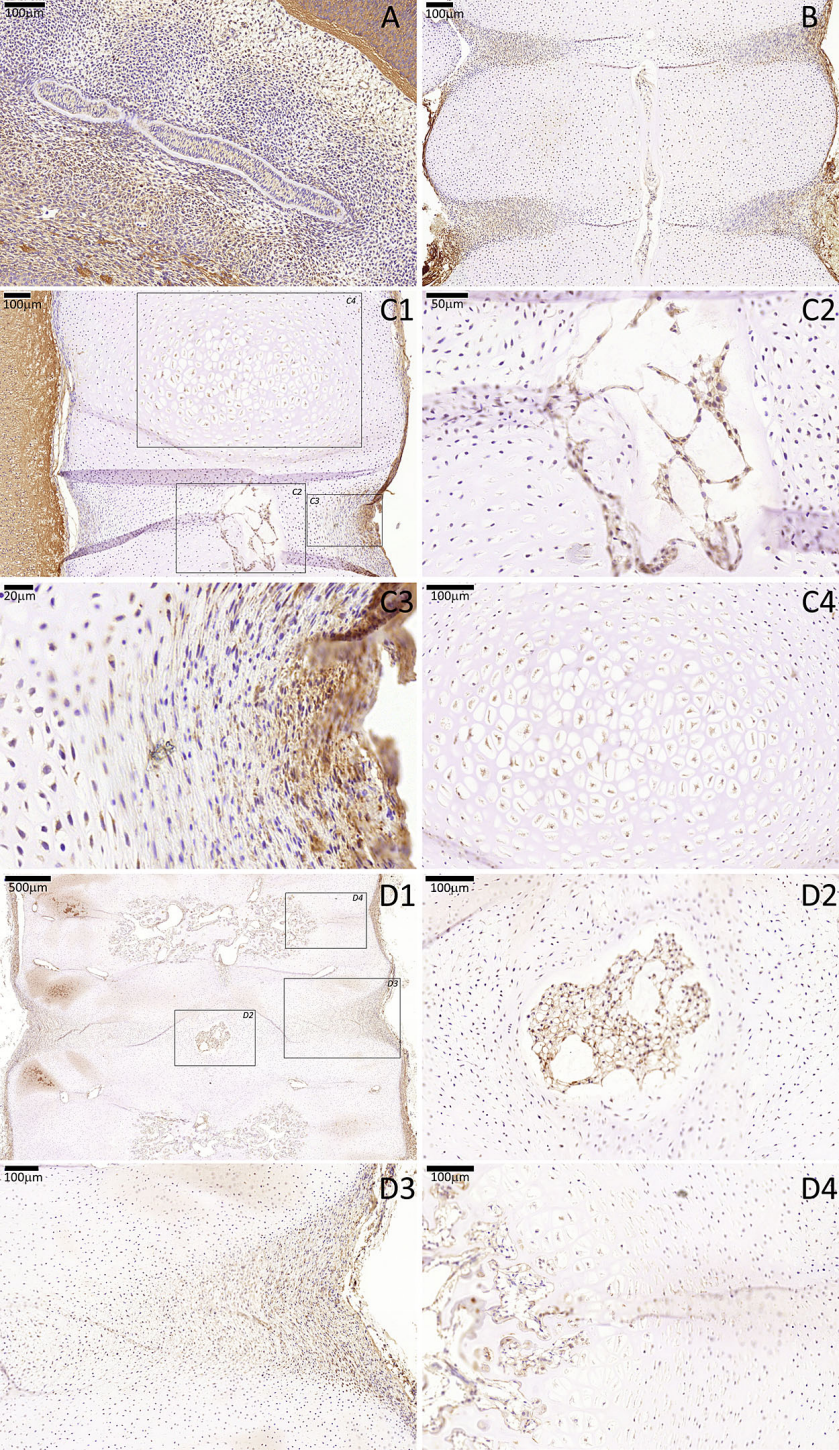
BASP1 immunostaining of a cohort of developing spines. BASP1 was localized to all notochordal and somite/sclerotomal cells at all stages analyzed, except at CS16, where no developing spine anlagen staining was found. (A) CS16 WPC, (B) 7.5 WPC, (C) 11 WPC, (D) 18 WPC. For stages 11 and 18 WPC, three higher magnifications are highlighted (squares), one that is centered to the developing notochordal NP (C2 and D2), other that is centered to the developing sclerotomal AF (C3 and D3) and another that is centered to the developing VB (C4 and D4).

BASP1 was expressed by all notochordal and somite cells in the 3.5 WPC specimen. In the 5.5 WPC specimen no BASP1 staining was seen in notochordal or sclerotomal cells. Between 6 and 18 WPC, BASP1 was co‐expressed by notochordal and sclerotomal cells (AF and VB anlagen); notochordal and sclerotomal staining, however, was weak between 6 and 8 WPC and became more intense between 9 and 18 WPC (Fig. 8).

#### Protein Markers Demonstrating No Detectable Expression in the Developing Spine

**Figure 9:**
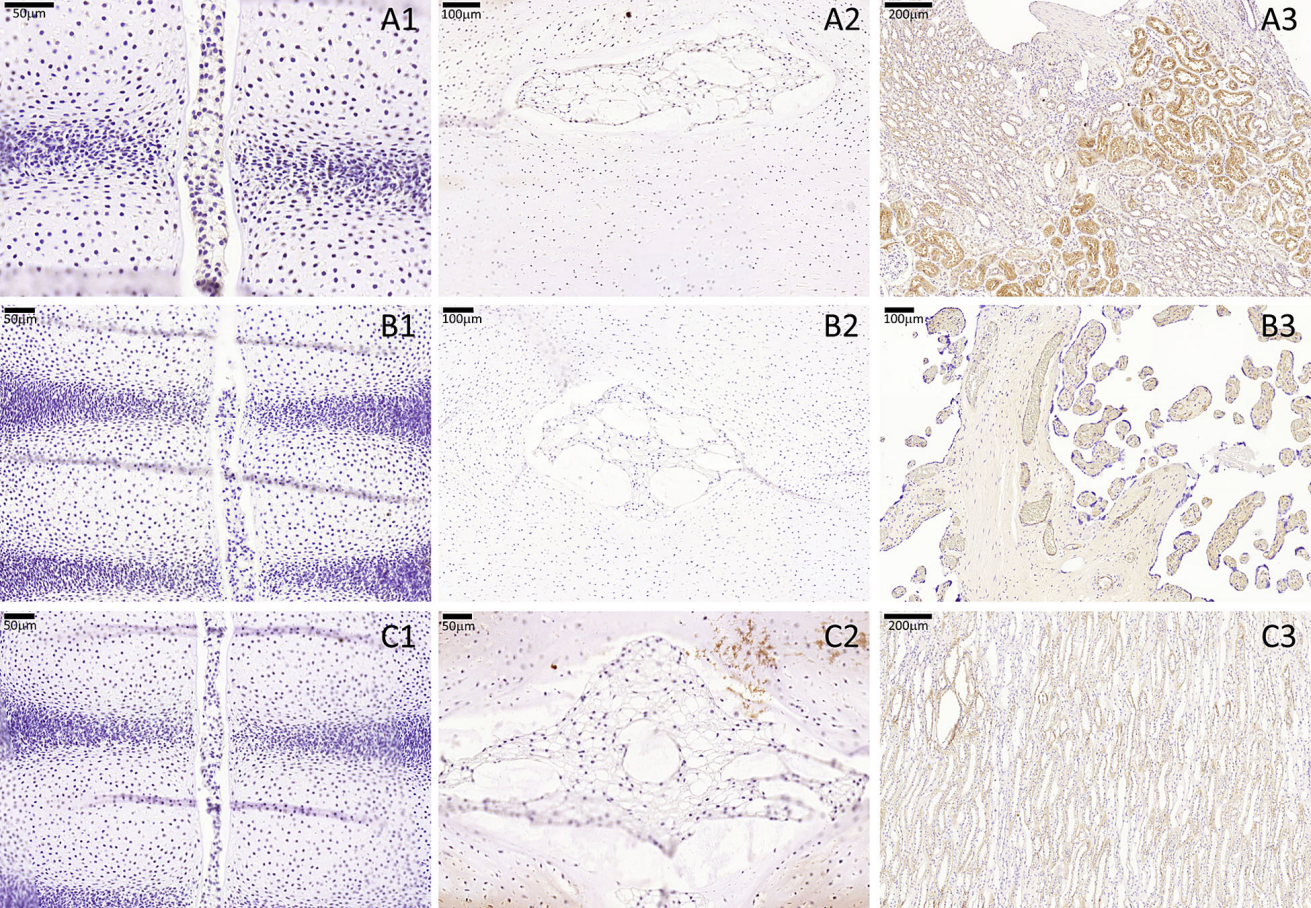
Negative developing spine markers. CD90, Tie2, and E‐Cad immunostaining was not seen in any developing spine anlagen at any of the stages analyzed. (A) CD90 immunostaining in specimens A1: CS16 and A2: 18 WPC, A3 depicts CD90 staining of kidney (positive control). (B) Tie2 immunostaining in specimens B1: CS16 and B2: 18 WPC. B3 depicts Tie2 staining of placenta (positive control). (C) E‐Cad immunostaining in specimens C1: CS16 and C2: 18 WPC. C3 depicts E‐Cad staining of kidney (positive control).

Tie2 was not expressed by any developing spine cell anlagen in any of the stages analyzed: Notochordal cells, sclerotomal AF anlagen cells, and sclerotomal VB anlagen cells were always negative for this protein. CD90 was not expressed by any developing spine cell anlagen in any of the stages analyzed: Notochordal cells, sclerotomal AF anlagen cells, and sclerotomal VB anlagen cells were always negative for this protein. E‐Cad was not expressed by any developing spine cell anlagen in any of the stages analyzed: Notochordal cells, sclerotomal AF anlagen cells, and sclerotomal VB anlagen cells were negative for this protein. Clear staining for each negative antibody was identified in positive control tissues (Fig. 9).

**Figure 10:**
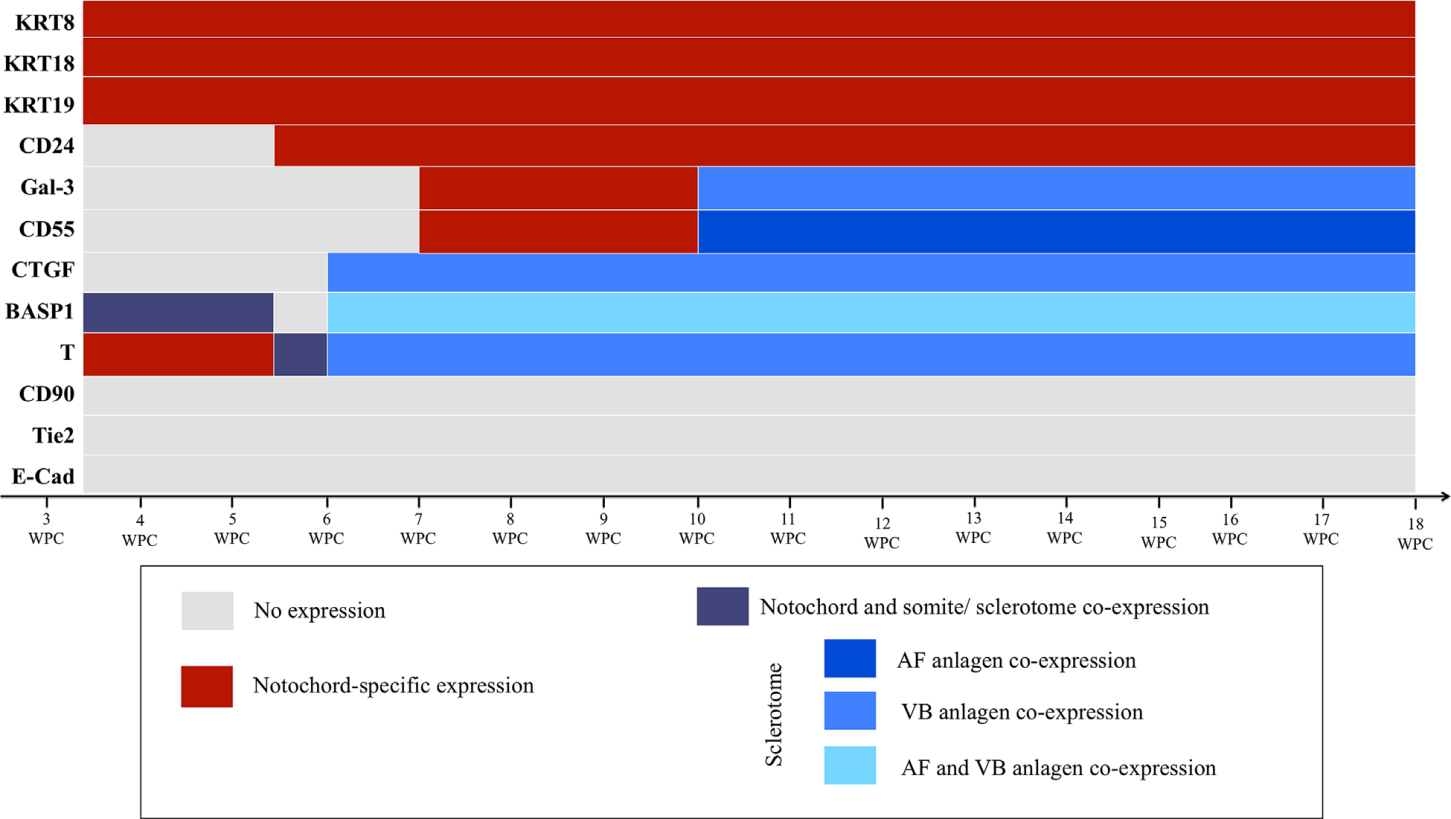
Schematic representation of the protein staining in the developing human spine. KRT8, KRT18, and KRT19 were notochord‐specific markers at all stages analyzed and CD24 was notochord‐specific between 5.5 and 18 WPC. The expression of GAL3, CD55, CTGF, BASP1, and T varied with development stage. CD90, Tie2, and E‐Cad were not expressed by any developing spine cell anlagen.

Figure 10 is a schematic illustration detailing areas of expression for each of the proteins analyzed.

## DISCUSSION

Studies investigating IVD degeneration and cell‐based therapies for its repair/regeneration have recently focused on understanding the native NP phenotype and the ontogeny of its cells. While studies in animals suggest that the adult NP is derived from the notochord,^1^, ^2^ due to an absence of cells with a distinct large‐vacuolated notochordal morphology in the adult human NP, the fate of notochordal cells in humans and the ontogeny of the cells populating the adult NP is not fully understood. This study aimed to assess the spatiotemporal expression of putative human notochordal cell markers in the embryonic and fetal human spine that could aid in elucidating the ontogeny of adult NP cells.

Thirty‐one human samples between the embryonic stages of 3.5–8 WPC (CS 10–23) and fetal stages of 8–18 WPC were used. This is, to the authors’ knowledge, the first description of the spatiotemporal variation in expression of putative notochordal cell markers in the developing human spine. The large number of samples utilized here allows for a detailed description of developing spine marker expression during the first and second trimesters of gestation.

There were no identifiable differences in marker staining between the IVD anlagens located in the cervical, thoracic, and lumbar regions possibly reflecting a common microenvironment to the different spinal regions at each developmental stage.

KRT8, KRT18, and KRT19 were found to be human notochord‐specific markers. Keratins are intermediate filaments mostly found in epithelial cells.^28^ Due to their unique cytoskeletal role, keratins act to provide structural integrity and they have also been found to regulate Fas‐mediated apoptosis and modulate cell size and protein synthesis.^29^ During the developmental stages analyzed, the notochord is the main axial element of the embryo and fetus, allowing the embryo to elongate.^30^ Later, its cells are subjected to continuous hydrostatic pressure^31^ exerted by the adjacent sclerotomal cells, leading to notochordal cell disappearance from the center of the developing VB to become localized in the central IVD. It is possible that, given the cytoskeletal properties of keratins, these proteins enable the notochord and its cells to exert their structural roles during these developmental stages. Furthermore, the expression of keratins by notochordal cells confirms the developing notochord as an epithelial‐like tissue, as previously suggested.^20^ Relevant to the IVD field, expression of keratins has also been found in the NP of immature rats,^5^ chondrodystrophic dogs,^7^ adult cows^8^, and adult humans, with and without histological features of degeneration.^10^, ^32^ Importantly, the expression of these notochordal markers by cells within the adult human NP indicates that, at least a subpopulation of adult NP cells is of notochordal ontogeny. The expression of these intermediate filaments in the NP of various animal species, at different stages of development, maturation, and disease and in cells with or without vacuoles and also, as shown here, in the human developing notochord, indicates that these proteins may be involved in a role that is intrinsic to all notochord‐derived NP cells.

This study also identified CD24 as a notochord‐specific marker between 5.5 and 18 WPC. CD24 is a cell surface protein that is expressed during B and T‐cell maturation and in differentiating neuroblasts.^33^ While some studies have linked it to cancer cell growth, proliferation, and survival,^34^ others have found it to be a marker of regenerative muscle cells^35^ and of hepatocyte^36^ and renal^37^ progenitor cells. During human development, CD24 is expressed by the intestinal mucosal, nasal, salivary gland, bronchial, and renal tubular epithelia and by hair follicles.^38^ In terms of IVD biology, CD24 has previously been identified in rat notochordal NP cells^4^, ^6^ and in human chordomas^4^ (tumors arising from remnants of the notochord), which corroborates the findings in this study confirming this protein as a notochordal marker. Additionally CD24 expression has been reported in discs from children and adolescents undergoing surgery for scoliosis.^23^ Furthermore, a subpopulation of CD24 positive mouse embryoid body cells showed spontaneous differentiation to cells with notochordal characteristics.^39^ Collectively, these findings have led to the proposal of CD24 as a healthy NP marker^40^ which may depict a cell of notochordal origin.

GAL3 and CD55 displayed an identical expression pattern: These markers were not expressed by any developing spine cell in the earlier stages, were notochord‐specific between 7 and 9 WPC and became co‐expressed by sclerotomal cells after 10 WPC. These findings contradict those of Oguz and colleagues who noted GAL3 expression in the NP and AF of the new‐born rat^41^ and with those of Leung and colleagues, who have proposed CD55 as a rat notochordal marker.^21^ Importantly, this further highlights the significant differences in marker expression between species.

BASP1 and CTGF were not notochord‐specific at any stage analyzed. BASP1, a membrane‐bound protein involved in nerve growth and synaptic plasticity previously identified in the NP but not in the AF of rat^6^ and bovine IVD^8^ was ubiquitously expressed in the developing spine at all stages analyzed except in the 5.5 WPC specimen. CTGF is a growth factor involved in extracellular matrix interactions that has been reported to be a key molecule in conditioned medium derived from canine^42^ and porcine notochordal cells.^43^ In this study, CTGF was co‐expressed by notochordal and VB anlagen cells after 6 WPC and until the latest stage analyzed (18 WPC). This supports the recent findings by Bedore and colleagues who identified the expression of this protein in the developing NP and VB (but also the AF) of embryonic mice, suggesting an important role of this protein in regulating IVD development.^13^

T is an embryonic transcription factor required for mesoderm formation and differentiation^44^ and notochord‐development.^45^ This protein has often been used as a marker of a notochordal phenotype.^46^, ^47^, ^48^ In our study, T was found to be expressed by all notochordal cells at all stages analyzed, which suggests that this transcription factor has a role in human notochordal cell development. However, its expression was not specific to notochordal cells, with developing sclerotomal AF anlagen cells between 5.5 and 18 WPC also expressing this marker and, therefore, making this marker unsuitable as a cell‐specific notochordal marker if used in isolation.

CD90, Tie2, and E‐Cad expression was not found in any developing spine anlagen cell at any of the developmental stages analyzed. CD90 has previously been proposed as a rat^6^ and immature human^23^ AF marker. Our findings do not support those observations, as this marker was not expressed by sclerotomal AF anlagen cells at any developmental stage. Sakai and colleagues have identified the presence of Tie2 positive (Tie2^+ve^) cells within NP cells from 8 to 10 week‐old mice and 18–69 year‐old humans and have found that Tie2^+ve^ cells, in culture, exhibited multipotency and self‐renewal capacity,^25^ although the ontogeny of such Tie2^+ve^ cells was not reported. Interestingly, in our cohort of samples, notochordal cells did not express Tie2, which suggests that the cells isolated by Sakai and colleagues may be of mesenchymal and not of notochordal origin. Additionally, E‐Cad, which has previously been identified in chordomas,^49^ was not expressed by developing human notochordal cells.

In summary, this study has, for the first time, described the spatiotemporal variation in expression of putative notochordal markers in the human developing spine, and has identified KRT8, KRT18, KRT19, and CD24 as human notochord‐specific markers and CD90, Tie2, and E‐Cad as negative spine markers. The fact that keratins, which have been identified in the human adult NP, were specific to the human developing notochordal cells indicates that the human adult NP has a population of notochord‐derived cells that have acquired a smaller non‐vacuolated morphology. This, therefore, suggests that at least a subpopulation of adult NP cells is notochord‐derived and that the adult NP consists of a heterogeneous population, as we have recently reported.^50^

While not a specific aim of this study, the identification of these positive and negative notochordal markers may also be helpful to understand the biology of chordomas.^4^, ^49^

Future studies may use these markers to separate human notochordal cells from sclerotomal cells and to produce a more thorough phenotypic and functional characterization of human notochordal cells. This will help in understanding the role of notochordal cells in human IVD development, maturation, degeneration, and regeneration. Furthermore, these markers may also be used to identify and isolate notochord‐derived cells from the human adult NP and to define phenotype in cell‐based tissue engineering studies.

## AUTHORS’ CONTRIBUTIONS

R.R.‐P. performed the experiments, analyzed, and interpreted the data, drafted the manuscript. A.B. planned the experiment, collected and processed samples, and reviewed the final manuscript draft. K.P.‐H. planned the experiment, collected and processed samples, and reviewed the final manuscript draft. N.H. planned the experiment, collected and processed samples, and reviewed the final manuscript draft. S.M.R. planned the experiment, interpreted the data, formulated manuscript outline, reviewed, and edited draft versions including final manuscript draft. J.A.H. planned the experiment, interpreted the data, formulated manuscript outline, reviewed, and edited draft versions including final manuscript draft. All authors have read and approved the final submitted article.

## Supporting information

Additional supporting information may be found in the online version of this article at the publisher's web‐site.

**Figure S1**. KRT8 immunostaining of a cohort of developing spines showing notochord‐specific expression of this marker.

**Figure S2**. KRT19 immunostaining of a cohort of developing spines showing notochord‐specific expression of this marker.
