# Supplementary figures and images for: Spatiotemporal analysis of putative notochordal cell markers reveals CD24 and keratins 8, 18, and 19 as notochord‐specific markers during early human intervertebral disc development
Source: J Orthop Res. 2016 Mar 7;34(8):1327–40. doi: 10.1002/jor.23205 (PMC5021113; doi:10.1002/jor.23205)

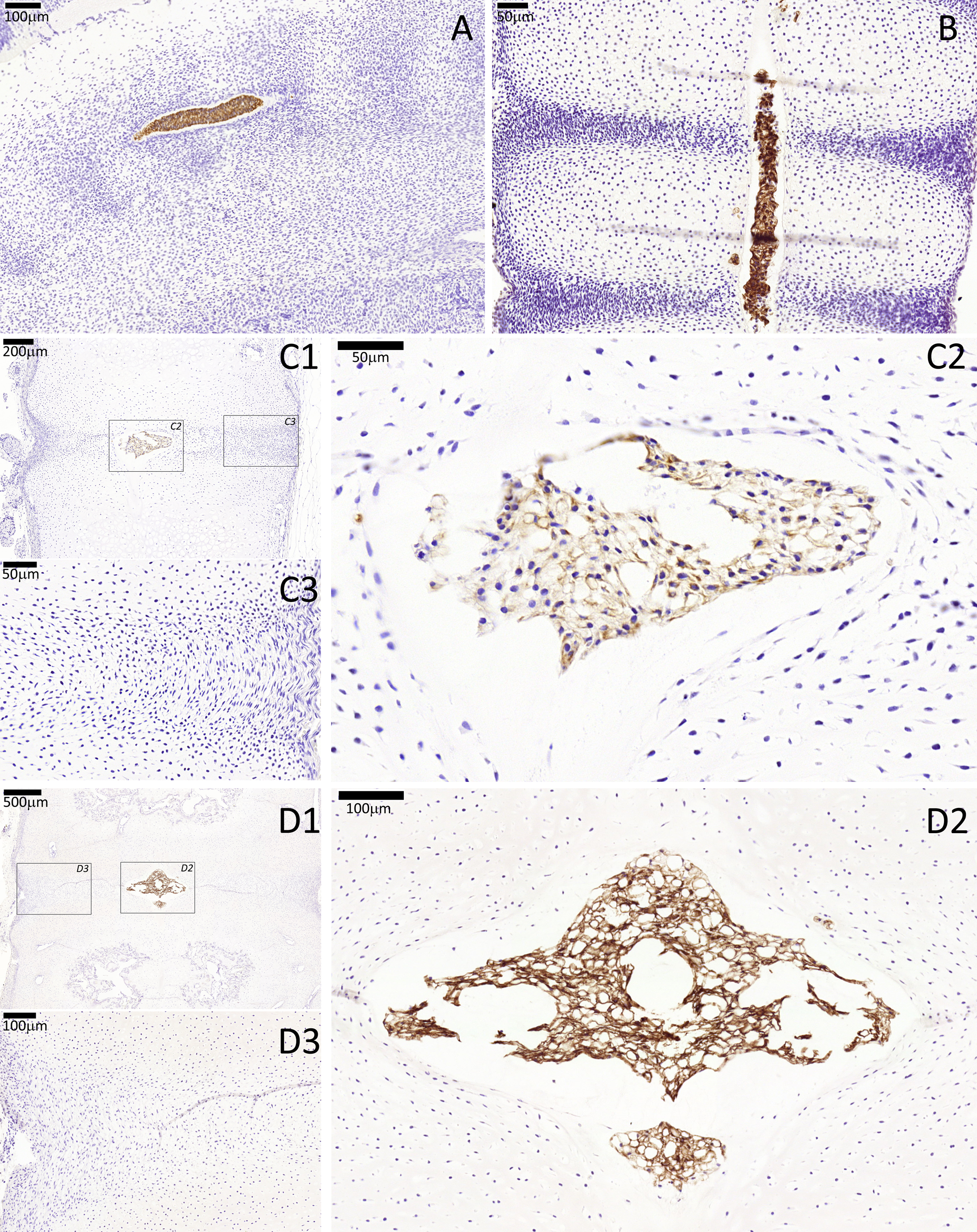

Supplement: Supplementary file 1 — Figure S1. KRT8 immunostaining of a cohort of developing spines showing notochord‐specific expression of this marker. [file JOR-34-1327-s001.tif]

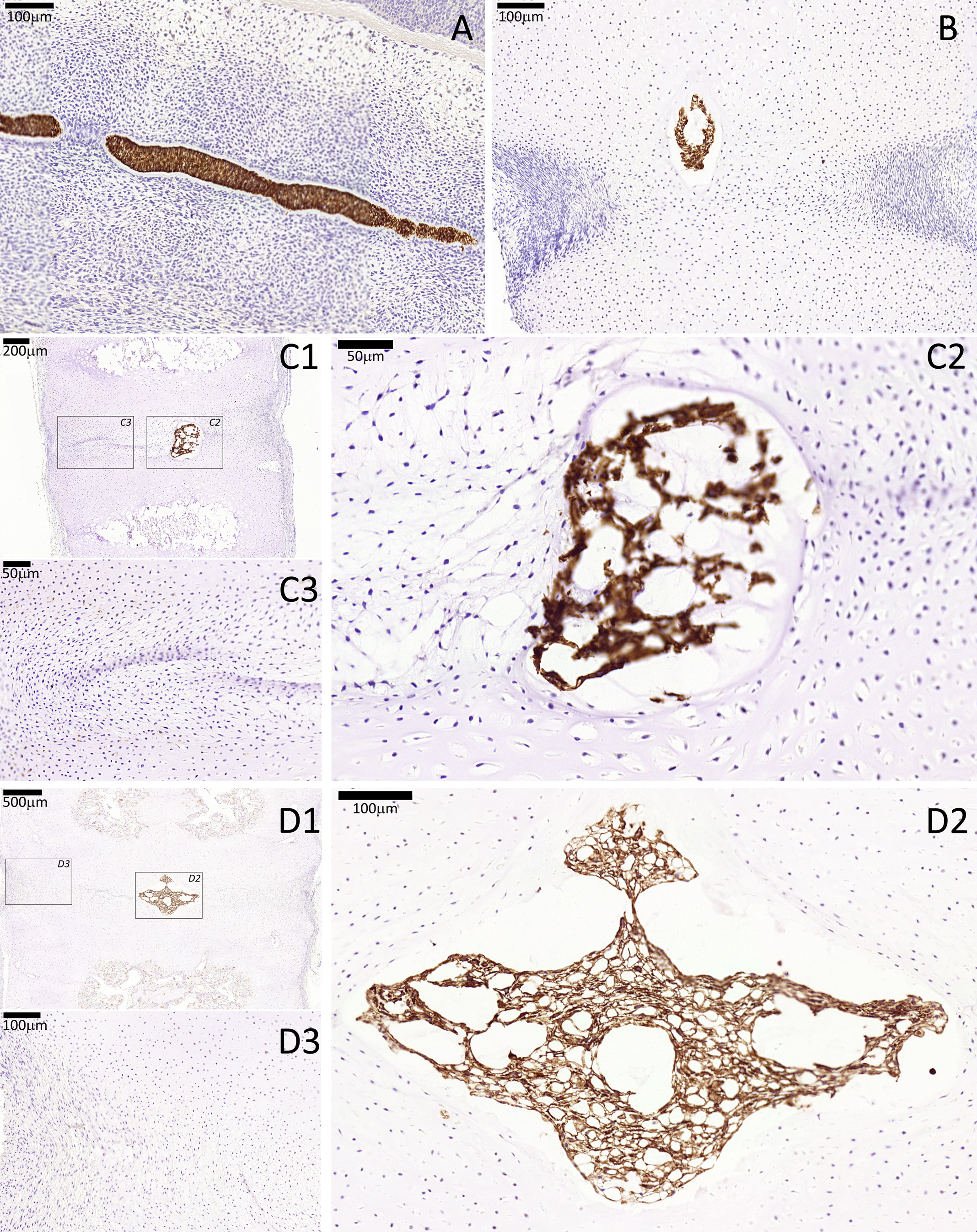

Supplement: Supplementary file 2 — Figure S2. KRT19 immunostaining of a cohort of developing spines showing notochord‐specific expression of this marker. [file JOR-34-1327-s002.tif]
